# Diabetes Prediction Using Feature Selection Algorithms and Boosting-Based Machine Learning Classifiers

**DOI:** 10.3390/diagnostics15202622

**Published:** 2025-10-17

**Authors:** Fatima Rahman, Sheyum Hossain, Jun-Jiat Tiang, Abdullah-Al Nahid

**Affiliations:** 1 Electronics and Communication Engineering Discipline, Khulna University, Khulna 9208, Bangladesh; 2 Centre for Wireless Technology, CoE for Intelligent Network, Faculty of Artificial Intelligence & Engineering, Multimedia University, Persiaran Multimedia, Cyberjaya 63100, Selangor, Malaysia

**Keywords:** diabetes prediction, feature selection algorithms (FSAs), machine learning, boosting classifier algorithms, medical diagnostics

## Abstract

**Background**: Diabetes mellitus is a significant primary global health concern that requires accurate diagnosis at an early stage to prevent severe complications. However, accurate prediction remains challenging due to limited, noisy, and imbalanced datasets. This study proposes a novel machine learning framework for improved diabetes prediction, addressing key challenges such as inadequate feature selection, class imbalance, and data preprocessing. **Methods**: This proposed work systematically evaluates five feature selection algorithms—Recursive Feature Elimination, Grey Wolf Optimizer, Particle Swarm Optimizer, Genetic Algorithm, and Boruta—using cross-validation and SHAP analysis to enhance feature interpretability. Classification is performed using two boosting algorithms: the light gradient boosting machine algorithm (LGBM) and the extreme gradient boosting algorithm (XGBoost). **Results**: The proposed framework, using the five most important features selected by the Boruta feature selection algorithm, outperformed other configurations with the LightGBM classifier, achieving an accuracy of 85.16%, an F1-score of 85.41%, and a 54.96% reduction in training time. **Conclusions**: Additionally, we have benchmarked our approach against recent studies and validated its effectiveness on both the Pima Indian Diabetes Dataset and the newly released DiaHealth dataset, demonstrating robust and accurate early diabetes detection across diverse clinical datasets. This approach offers a cost-effective, interpretable, and clinically relevant solution for early diabetes detection by reducing the number of input features, providing transparent feature importance, and achieving high predictive accuracy with efficient model training.

## 1. Introduction

Diabetes is a chronic metabolic disorder that occurs when the glucose level of the blood increases and the body cannot produce or use insulin effectively [1]. When insulin production is insufficient or cells become resistant to insulin, glucose accumulates in the bloodstream. This steady high blood glucose level causes severe health complications, including nerve damage, kidney failure, vision loss, cardiovascular diseases, and, in some extreme cases, can ultimately result in premature death. Among 40 different types of diabetes, the most common forms are Type 1 Diabetes, Type 2 Diabetes, gestational diabetes, and pre-diabetes [2,3]. However, all of these types lead to serious complications if left unmanaged and untreated. In one study, it has been shown that approximately 5.5% of the global population is affected by diabetes, with a prediction that it will rise by nearly 48% in the coming years [4]. In the United States alone, 34.3 million people, which is about 10.5% of the total population, were living with diabetes in 2018 [5]. This deadly disease significantly increases the risk of heart disease, microvascular complications, and premature death [6]. According to the World Health Organization (WHO), in 2019, diabetes was responsible for an estimated 1.9 million deaths globally and remains a leading cause of mortality worldwide [7]. A study by the International Diabetes Federation (IDF) shows that the number of people living with diabetes will reach 783 million by 2045 if the present trends of its spread continue [8]. Therefore, it is crucial to ensure early detection and intervention in patients with diabetes in order to reduce their burden and suffering and improve their conditions.

Early diagnosis of diabetes can prevent complications and increase the chances of saving lives and reducing costs. However, research findings highlight that widespread diabetes screening is expensive, time-consuming, and can overwhelm healthcare infrastructures, especially in resource-limited settings [9]. Evidence reveals that traditional statistical approaches for diabetes risk prediction, such as logistic regression, have been widely used previously but often fail to capture complex, nonlinear relationships inherent in biological and lifestyle data [10]. Furthermore, manual diagnosis relies heavily on the subjective judgment of healthcare professionals, which can sometimes lead to errors [11]. Therefore, it is evident that there is a pressing need for efficient, accurate, and cost-effective diagnostic tools that can automatically detect early diabetes risks while reducing the burden on healthcare providers. Recently, advances in machine learning (ML) and artificial intelligence (AI) have offered promising alternatives for diabetes risk prediction. These models excel at processing large, complex datasets and identifying patterns that may not be accurately acquired through traditional analysis [12,13]. There are some prominent datasets that are commonly used for studies and research works in this field. Using these conventional diabetes datasets, such as the PIMA Indians Diabetes Dataset (PIDD), numerous researchers have attempted to construct a system for the detection of diabetes using advanced ML and deep learning (DL) approaches [14]. A comprehensive study revealed that out of 107 studies, the majority of researchers used publicly obtainable datasets. Among them, PIDD was used the most in the studies, being used 13 times, and there were some other datasets, such as the HRF Image Dataset, that were used only once [15].

According to some recent studies [16,17,18,19], several machine learning models have been implemented for diabetes prediction, but still some key gaps remain. Most studies neglect proper feature selection or rely on all features, which increases overfitting, cost, and complexity, and reduces interpretability. Furthermore, class imbalance is often overlooked, which may cause the models to be biased toward the majority class and lower sensitivity. Additionally, inadequate preprocessing, such as missing value imputation, outlier detection, and class balancing, further weakens robustness and generalizability. Without addressing these issues, models risk becoming less accurate, trustworthy and practical for clinical use.

To address these issues, our study proposes an integrated machine learning framework that tackles these critical limitations. Specifically, our framework combines advanced feature selection algorithms with thorough data preprocessing and class balancing techniques to build an accurate, efficient, and interpretable diabetes prediction model that can achieve better performance with even a smaller number of features used. The Pima Indian Diabetes Dataset (PIDD) is chosen as the primary dataset of this study due to its widespread use and benchmarking value in diabetes prediction research. To further evaluate the robustness and generalizability of our proposed framework, we also validated it on the DiaHealth dataset, a recently developed Bangladeshi cohort comprising 5437 patients with 14 attributes, including demographics, vital signs, clinical parameters, and family history of comorbidities. This additional dataset not only provides more abundant and more diverse clinical information but also allows us to assess the applicability of our approach in a real-world, large-scale setting. We have selected boosting algorithms as the core classifiers because of their modern capabilities in handling imbalanced datasets via built-in regularization and flexible loss functions.

Contributions of this research:

▪ Developed a robust preprocessing pipeline, including mean imputation, outlier removal using the interquartile range (IQR) method, and oversampling, enhancing data quality and ensuring reliable model performance.
▪ Systematically evaluated five feature selection algorithms—Recursive Feature Elimination (RFE), Grey Wolf Optimizer (GWO), Particle Swarm Optimizer (PSO), Genetic Algorithm (GA), and Boruta—and validated feature importance with SHAP analysis, resulting in an interpretable and clinically meaningful feature set.
▪ Demonstrated that LightGBM combined with the features selected by the Boruta algorithm achieves superior predictive performance (accuracy: 85.16%, F1-score: 85.41%) while reducing feature dimensionality from 8 to 5, highlighting both efficiency and model robustness.
▪ Reduced model training time by nearly 55% through feature reduction, showing that the framework improves computational efficiency without compromising predictive accuracy.
▪ Reducing the number of features lowers computational cost and accelerates model training and inference, making the framework a cost-effective and practical tool for early diabetes detection and faster clinical decision-making.
▪ Validated the framework on an independent, recently released clinical dataset (DiaHealth), demonstrating robustness and generalizability. This validation shows its potential for reliable early diabetes prediction in novel clinical datasets with limited prior studies.

## 2. Related Works

Recent progress in machine learning (ML) has enhanced both the accuracy and interpretability of medical diagnostic models, supporting more effective healthcare delivery. According to a research work [15], Naïve Bayes with K-medoids undersampling achieved 79% receiver operating characteristic (ROC) performance and revealed a positive correlation between high HDL levels and diabetes onset. This work highlights the value of sampling strategies in handling class imbalance and uncovering clinical correlations. In Ref. [17], traditional classifiers such as decision tree (DT), Support Vector Machine (SVM), and Naïve Bayes (NB) were evaluated on the Pima Indian Diabetes Dataset (PIDD), with NB showing superior accuracy of 76.30%. This comparative study established a baseline understanding of classical classifiers for diabetes prediction. In a similar approach, ref. [18] demonstrated that Random Forests (RF) provided better diagnostic performance with an error rate of just 0.21 using 40 trees on the same dataset. Further enhancing predictive accuracy, ref. [19] applied multiple classifiers, including SVM, RF, NB, DT, and KNN, on a diabetes dataset from the UCI repository, which identified NB as the top performer with an accuracy of 82.30% using 11 out of 15 features. Feature selection was the focus of [20], where a rough set-based ensemble approach combined with RF achieved 73.04% accuracy using only 3 out of 8 features, showing the importance of optimal feature subsets. Similarly, numerous works have been conducted in this field, with some of the key findings presented in Table 1.

While these studies provide valuable insights, they often lack comprehensive feature selection, class imbalance handling, and interpretability. To address these gaps, the present work proposes a robust and efficient ML framework, which is described in the following section.

## 3. Methodology

**Figure 1:**
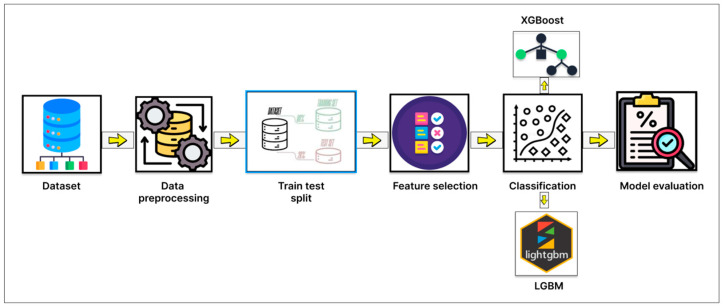
Diagram of the overall methodology of this study.

This section presents a step-by-step workflow of our proposed diabetes prediction framework, which reduces the number of features using the Pima Indian Diabetes Dataset (PIDD) as the primary dataset. The overall pipeline includes dataset preparation, robust preprocessing, and the implementation of 5 feature selection algorithms with 5-fold cross-validation to identify the most relevant input features. To enhance the interpretability and clinical relevance of the selected features, we also applied SHAP (Shapley Additive exPlanations) analysis. Finally, we trained and evaluated using machine learning classifiers, focusing on boosting-based models, using the selected features. To visualize the steps, the diagram of our methodology is given below in Figure 1.

### 3.1. Dataset Description

#### 3.1.1. Pima Indian Diabetes Dataset (PIDD)

The primary dataset used in this study is the Pima Indian Diabetes Dataset (PIDD) [29]. This publicly available dataset has been widely utilized in machine learning research for the prediction of Type 2 Diabetes due to its relevance and benchmarking utility. The dataset consists of 768 instances in total, each of whichrepresents a female patient of Pima Indian heritage, aged 21 years or older. Among these, 268 patients (34.9%) are diabetic and 500 (65.1%) are non-diabetic, indicating a moderate class imbalance. Table 2 presents attribute information along with their corresponding measures, including the record count, minimum (min) value, maximum (max) value, mean and standard deviation (std).

**Figure 2:**
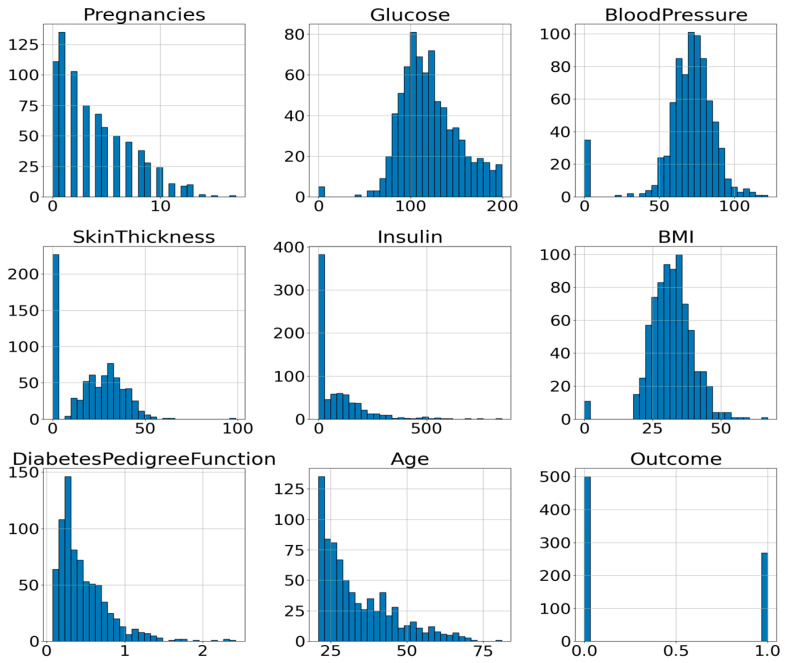
Histogram of the Attributes of PIDD.

To gain a better understanding of the attribute’s distribution, its histogram can be plotted. A histogram is a valuable tool for visualizing and understanding the distribution of data samples within a dataset. It offers a reliable approximation of any probability density with minimal assumptions [30]. Figure 2 illustrates the histograms of all input attributes (excluding the outcome variable), showing the frequency distribution of values across each feature’s range.

**Figure 3:**
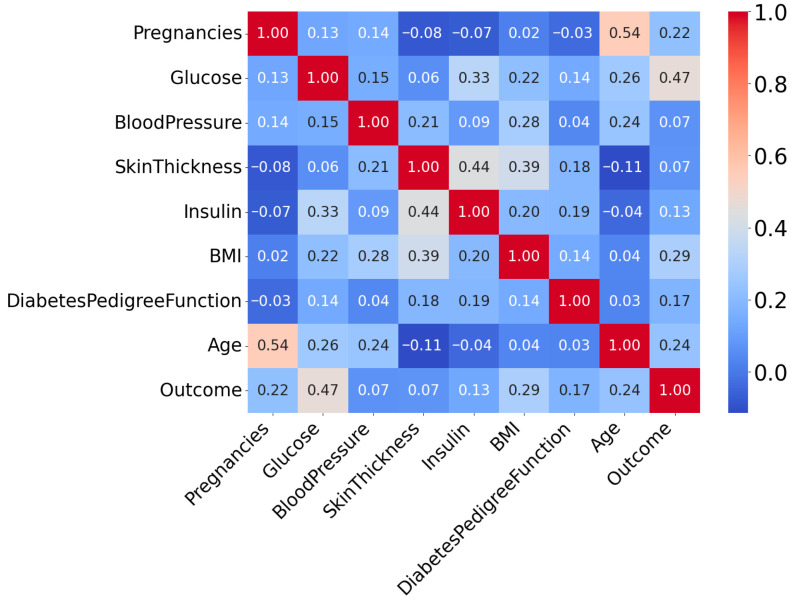
Correlation Heatmap of Attributes of PIDD.

The interrelationships among the dataset’s attributes are analyzed using Correlation Coefficient Analysis (CCA). Generally, it is used to determine the degree of association [31]. Figure 3 presents the correlation heatmap of all input variables used for diabetes prediction.

From this correlation heatmap, it is evident that the correlation between variables is not very strong. Here, the “outcome” attribute is the target column. The target variable is binary, where 1 indicates the Patient is diabetes-positive and 0 represents the Patient is diabetes-negative.

#### 3.1.2. DiaHealth: A Bangladeshi Dataset for Type 2 Diabetes Prediction

**Figure 4:**
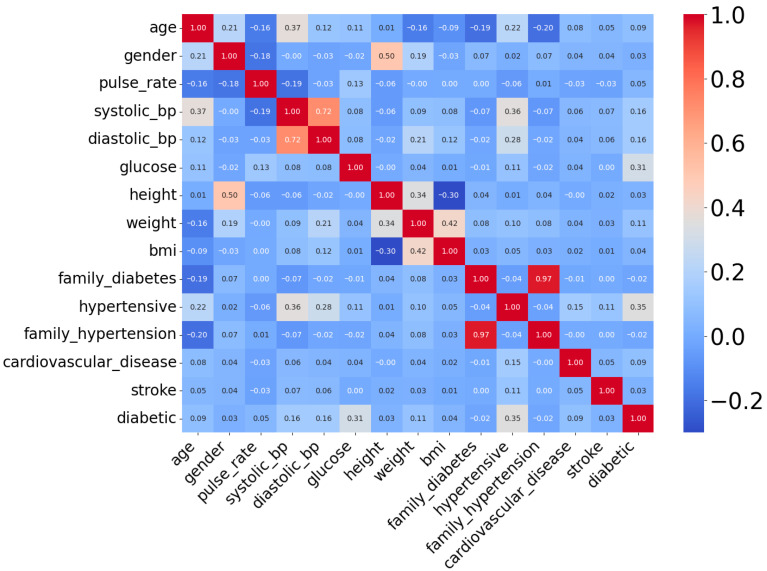
Correlation heatmap of attributes of DiaHealth dataset.

The DiaHealth dataset, titled “DiaHealth: A Bangladeshi Dataset for Type 2 Diabetes Prediction”, is a comprehensive and high-quality clinical resource comprising medical and demographic records of 5437 patients [32]. It was specifically designed to support the development and evaluation of machine learning models for the detection, prediction, and management of Type 2 Diabetes (T2D). The dataset includes 14 independent features that capture a broad spectrum of patient information relevant to diabetes diagnosis. These features span demographics (age, gender), physiological and clinical parameters (pulse rate, systolic and diastolic blood pressure, glucose level, body mass index, cholesterol level), lifestyle factors (smoking history, physical activity, dietary habits), and medical history (hypertension, cardiovascular disease, family history of diabetes). Each record is labeled with a binary outcome variable, indicating whether the Patient is diabetic (positive class) or non-diabetic (negative class). The dataset is particularly valuable because it not only includes widely recognized diabetes indicators such as glucose and BMI but also integrates comorbidity-related variables (e.g., hypertension and cardiovascular disease), making it more representative of real-world clinical populations than traditional benchmark datasets. Table 3 provides a detailed overview of the features, their data types, and descriptions, while Figure 4 presents the correlation analysis between features and the target variable. Together, these characteristics make DiaHealth a suitable secondary dataset for evaluating the generalizability of the proposed framework beyond the Pima Indian Diabetes Dataset (PIDD).

### 3.2. Data Preprocessing

Before applying machine learning algorithms, the dataset must be preprocessed, as the presence of missing data in features can significantly affect model performance. Table 4 shows the number of missing values per feature in the dataset.

From Table 4, there are five features (represented by five columns) with missing valuesthat need to be handled. One of the most widely used methods is preprocessing through imputation, which involves estimating and filling in the missing data [33]. In this study, mean imputation was used to handle missing data by replacing each missing value with the mean of the corresponding feature’s non-missing values. This method is preferred due to its simplicity and the fact that it preserves the original sample size.

After mean imputation in this research, the interquartile range (IQR) method was employed to detect and remove outliers from the dataset. The IQR [34] method is a robust statistical technique commonly used for outlier detection because it effectively represents the data spread without assuming a normal distribution. The steps of this process are given below:

1. The dataset is first sorted in ascending order for each numeric feature.
2. The first quartile (Q1), which is the median of the lower half of the data, and the third quartile (Q3), the median of the upper half, are calculated.
3. The IQR is computed as the difference between Q3 and Q1 (IQR = Q3 − Q1).
4. Outliers are defined as any data points lying below Q1 − (1.5 × IQR) or above Q3 + (1.5 × IQR)
5. These outliers are removed from the dataset to improve data quality.

**Figure 5:**
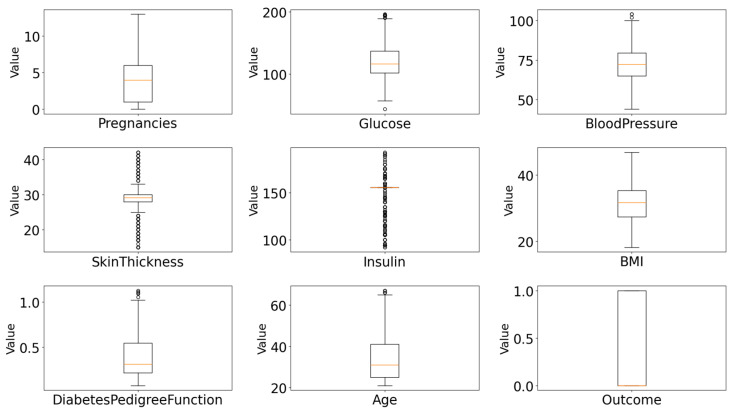
Feature distributions of all numeric attributes after IQR-based outlier removal and mean imputation.

The algorithm iterates through all numerical features in the dataset, calculates the bounds for acceptable values based on the IQR rule, and filters out rows containing outlier values in any numeric feature. This ensures a clean dataset free of extreme values that could bias the machine learning model. This substantial reduction indicates the presence of many extreme values that could have negatively impacted model performance. The distribution of features after outlier cleaning are given in Figure 5.

Even after the outline cleaning, there is a noticeable imbalance in the dataset, where the majority class (Outcome = 0) consisted of 302 samples, while the minority class (Outcome = 1) had only 160 samples. This imbalance can negatively impact the performance of machine learning models, especially in correctly identifying instances of the minority class. To address this issue, a random oversampling technique was applied. This method was selected over other sampling strategies due to its simplicity, efficiency, and strong empirical performance on imbalanced datasets [25]. In this case, it increased the number of class 1 samples from 160 to 302, thereby equalizing the class counts. The preprocessing pipeline was carefully designed to ensure model robustness and handle real-world data imperfections.

After the preprocessing stage, the dataset needs to be split into training and test parts. Various train-test split ratios, such as 60:40, 50:50, and 70:30, are commonly used in data processing [35]. In this work, 70% of the data was used for training and 30% for testing, ensuring that the model was trained and evaluated on separate, representative subsets of the dataset.

### 3.3. Feature Selection

Feature selection is the mechanism of identifying a subset of relevant features from the original dataset based on specific criteria defined by mathematical or statistical models. Selecting features with higher predictive value is essential for enhancing the accuracy, robustness and generalizability of classification algorithms. This study consists of two parts in feature selection. They are as follows:

▪ Using SHAP analysis;
▪ Using Feature selection algorithms.

**SHAP feature selection**

SHAP (Shapley Additive exPlanations) is a widely used method in Explainable Artificial Intelligence (XAI) that provides transparent insights into machine learning models by quantifying the contribution of each feature to individual predictions [36]. By integrating SHAP at this stage, the study ensured that selected features were not only statistically relevant but also interpretable, offering transparency early in the pipeline.

**Feature selection algorithms**

Feature selection algorithms (FSAs) can be broadly categorized into three main types: Filter, Wrapper, and Embedded methods [37]. Among them, Wrapper methods themselves can be further subdivided into deterministic and metaheuristic approaches. While deterministic Wrappers rely on systematic, rule-based search strategies, metaheuristic algorithms (MHAs) employ stochastic, nature-inspired global search techniques that overcome limitations of deterministic methods.

**Choosing of feature selection algorithm and justification**

According to previous studies, the Genetic Algorithm exhibits strong capabilities and high prediction efficiency in early-stage diabetes risk prediction [38]. Another research work revealed that a Genetic Algorithm and a decision tree were used for the Classification of Diabetes Mellitus on the Pima Indians Diabetes Dataset (PIDD), achieving a remarkable accuracy of 82.1256% with selected features [39]. This study showed how effectively GA can be used to reduce error rate to help in the decision-making process. In Ref. [40], two approaches were implemented to compare performance in accurately detecting diabetes. The first approach entailed classification using Logistic Regression, K-Nearest Neighbor (KNN), ID3 decision tree, C4.5 decision tree, and Naïve Bayes algorithms. The second approach employed Principal Component Analysis (PCA) and Particle Swarm Optimization (PSO) for feature reduction before applying the same classification methods used in the first approach. A comparative analysis was conducted, and the results clearly demonstrated that the second, feature-reduction-based approach achieved higher accuracy and lower computation time, indicating its efficiency over the traditional classification approach. A separate investigation showed that a novel wrapper-based feature selection utilizing Grey Wolf Optimization (GWO) and an Adaptive Particle Swam Optimization (APSO) was performed to optimize the Multilayer Perceptron (MLP) to reduce the number of required features, where GWO achieved higher prediction accuracy [41]. A related study compared the performance of five alternative algorithms employing the Recursive Feature Elimination (RFE) feature selection method and resulted in higher performances when using the selected feature set rather than all features [42]. According to another study using Boruta feature selection and ensemble learning [43], the result showed that this feature selection performed better.

**Figure 6:**
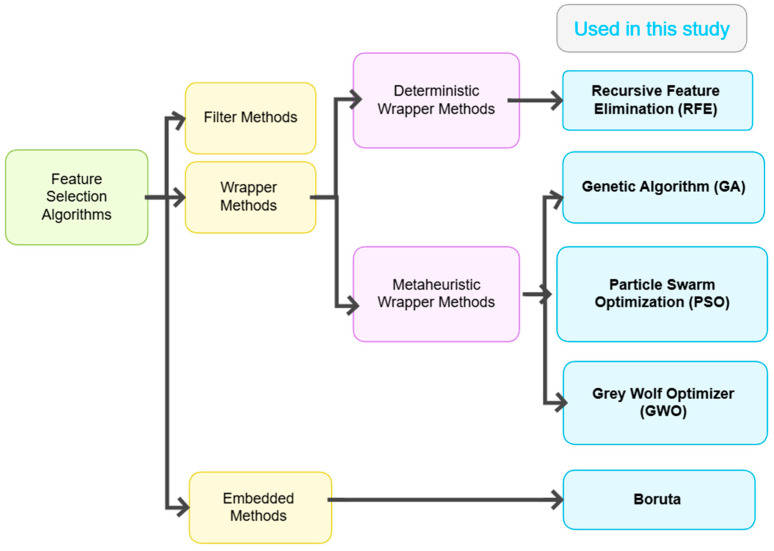
Classification of feature selection algorithms.

Considering this, five feature selection algorithms were employed in this study: Genetic Algorithm (GA), Particle Swarm Optimization (PSO), Grey Wolf Optimizer (GWO), Recursive Feature Elimination (RFE), and Boruta. These algorithms were carefully selected to represent a diverse and complementary set of approaches. These five algorithms were chosen based on their proven success in high-dimensional feature selection tasks, particularly in medical and diagnostic domains, and their ability to handle complex, nonlinear feature interactions that are common in health-related data. Filter methods were intentionally excluded, as they assess features independently of the learning algorithm and often fail to capture interactions that influence prediction performance. The classification is presented as a tree diagram in Figure 6, which illustrates the algorithms used.

Each feature selection method was evaluated using 5-fold cross-validation to ensure robustness and reduce the risk of overfitting. For each algorithm, the most frequent features were selected as the optimal features from the cross-validation sets. These features were then used to train the final classification models on a 70:30 train-test split. Model performance was assessed using standard evaluation metrics, including accuracy, recall, and F1-score.

**Recursive Feature Elimination (RFE)**

Recursive Feature Elimination (RFE) [44] is a wrapper-based feature selection algorithm that aims to identify the most relevant subset of features for a given predictive task. RFE operates by recursively training a model, ranking features based on their importance, and then eliminating the least important ones. This process continues until the specified number of features is reached. However, the choice of how many features to retain can significantly affect model performance.

**Genetic Algorithm (GA)**

Among various optimization-based methods, Genetic Algorithm (GA) [45] has gained wide usage for its ability to search large feature spaces using principles inspired by biological evolution. GA is a population-based stochastic search technique that mimics natural selection, crossover, and mutation processes to evolve better feature subsets. Its flexibility allows the application to complex, non-differentiable, or high-dimensional spaces without requiring prior knowledge of the domain.

**Particle Swarm Optimization (PSO)**

Particle Swarm Optimization (PSO) [46] is a well-established population-based metaheuristic algorithm inspired by the social behavior of birds flocking or fish schooling. In the context of feature selection, each particle in the swarm represents a binary vector indicating a subset of selected features. The swarm explores the feature space by adjusting particle positions based on both personal experience and social influence, with the goal of optimizing a fitness function (e.g., classification accuracy).

**Grey Wolf Optimizer (GWO)**

Grey Wolf Optimizer (GWO) [47] is a nature-inspired, population-based metaheuristic that mimics the leadership hierarchy and cooperative hunting strategy of grey wolves in the wild. In the context of feature selection, each grey wolf represents a binary vector encoding a subset of features. The binary encoding enables direct application to high-dimensional feature selection tasks.

**Boruta algorithm**

Boruta [48] is an all-relevant feature selection algorithm that identifies both strongly and weakly relevant features in relation to the target variable. Unlike traditional minimal-optimal approaches that focus only on minimizing error, Boruta retains all attributes that carry predictive value. It functions as a Wrapper method around Random Forest and leverages the model’s feature importance to compare real features with randomized “shadow” versions, filtering out irrelevant ones. Boruta’s strength lies in its robustness to noise, suitability for complex data, and ability to uncover subtle patterns—especially useful in biomedical domains.

**Cross-validation**

Cross-validation is a widely used data resampling technique for model selection and evaluation in statistical and machine learning tasks [49]. It helps in tuning hyperparameters, preventing overfitting, comparing algorithms, and estimating generalization error. Among its variants, k-fold cross-validation is one of the most common. In this method, the dataset is randomly divided into k disjoint subsets (folds) of roughly equal size. Each fold serves once as the validation set, while the remaining k−1 folds are used for training. This process is repeated k times, and the model’s performance is averaged over all k iterations to estimate its cross-validated performance.

### 3.4. Machine Learning Models for Classification

From Table 1, it has been shown that several classification algorithms have been implemented [16,17,18,19,20] by researchers to achieve better performances. But statistics showed that, the LGBM model outperformed KNN, NB, Bagging, SVM, and RF in the case of the ZMHDD dataset [50]. Another study demonstrated that XGBoost significantly outperformed other methods in this type of classification [21]. Additionally, another study revealed that a hybrid of boosting algorithms also achieved excellent performance [51]. That is why LGBM and XGBM were chosen as classifier algorithms in this study. LGBM and XGBoost were selected as the core classifiers for this study due to their superior handling of structured/tabular data, built-in mechanisms for managing missing values, and high interpretability through feature importance analysis. Both models support regularization and gradient boosting, which help avoid overfitting and improve generalization on imbalanced datasets, a common challenge in medical prediction tasks.

**Light Gradient Boosting Machine (LGBM) algorithm:**

Developed by Microsoft in 2016, LGBM is a fast, distributed, and open-source gradient boosting framework designed for high performance and efficiency. It is actually an improved version of the gradient boosting machine algorithm [52]. It employs a histogram-based algorithm to accelerate training and reduce memory consumption, making it highly suitable for large datasets and resource-constrained environments such as Low Power Lossy Networks (LLNs). LGBM incorporates two key techniques: Gradient-based One-Side Sampling (GOSS) and Exclusive Feature Bundling (EFB), which address the limitations of traditional histogram-based methods used in gradient boosting decision tree (GBDT) frameworks. The mathematical analysis in GOSS is presented below in Equation (1).

(1) $$\hat{V_{j}}(d)=\frac{1}{n}(\frac{({\sum_{x_{i}{\in A}_{l}}g_{i}+\frac{1-a}{b}\sum_{x_{i}{\in B}_{l}}g_{i})}^{2}}{n_{l}^{j}(d)}+\frac{({\sum_{x_{i}{\in A}_{r}}g_{i}+\frac{1-a}{b}\sum_{x_{i}{\in B}_{r}}g_{i})}^{2}}{n_{r}^{j}(d)})$$

where

$\hat{V_{j}}(d)$ = estimated variance gain over the subset A ∪ B;
$A_{l}$ = {$x_{i}\;\in A\;:\;x_{ij}\;\leq d$}, $A_{r}$ = {$x_{i}\;\in A\;:\;x_{ij}>d$};
$B_{l}$ = {$x_{i}\;\in B\;:\;x_{ij}\;\leq d$}, $B_{r}$ = {$x_{i}\;\in B\;:\;x_{ij}>d$};
*n* = total number of training samples, $g_{i}$ = gradient of the loss;
A = subset of samples with largest |gradients|, B = subset of randomly sampled remaining samples, a = top fraction of samples with largest gradients, b = fraction of remaining samples randomly sampled and $\frac{1-a}{b}$ is the coefficient.

GOSS selectively retains instances with larger gradients (which contribute more to the information gain) while randomly dropping instances with smaller gradients, thereby preserving the accuracy of split point estimation while reducing computational load. Meanwhile, EFB reduces model complexity by bundling mutually exclusive features into a single feature, further enhancing training efficiency. The model uses a logistic regression loss function to optimize classification, which penalizes discrepancies between predicted probabilities and accurate labels, effectively calibrating the detector and estimating prediction uncertainty. The loss function is given in Equation (2).

(2) $$Logloss=-\frac{1}{N}\sum_{i=i}^{N}y_{i}log\left(\hat{y}\right)+(1-y_{i})log(1-\hat{y})$$

where i denotes the given observation, $y_{i}$ denotes the actual value, and $y_{i}$ represents the probability of prediction.

**Extreme Gradient Boosting (XGBoost):**

It is a scalable, high-performance machine learning algorithm widely used for classification tasks. It is based on the gradient boosting framework, which constructs an ensemble of weak learners, typically decision trees, by optimizing a loss function in an additive manner. XGBoost [53] is known for its robustness, speed, and ability to handle sparse data and missing values efficiently.

The strength of XGBoost lies in its regularization mechanisms and hyperparameter flexibility. Parameters such as *n*_estimators, learning_rate, max_depth, and min_child_weight allow fine control over model complexity and performance. The algorithm integrates a regularized objective function that combines the training loss with a penalty term to discourage overfitting. XGB is presented by the following Equation (3).

(3) $$\hat{y_{i}}=\phi \left(x_{i}\right)=\sum_{k=1}^{K}f_{k}\left(x_{i}\right),\;f_{k}\in F$$

Here $f_{k}$ is the non-dependent decision tree, F is area regression trees, K are the additive functions and $x_{i}$ denotes the variables with no dependencies. And the loss function, denoted by L(ϕ). Its equation is given in Equation (4).

(4) $$L(\phi )=\sum_{i}l(\hat{y_{i}},\;y_{i})+\sum_{k}P(f_{k})$$

Here, l($\hat{y_{i}}$, $y_{i}$) measures the difference between the predicted and actual values, while the penalty term P accounts for the number of leaves and their weights in the decision trees.

### 3.5. Performance Analysis

To evaluate the performance of the classification models developed in this study, several standard metrics were utilized, including accuracy, precision, recall, and F1-score, to examine the distribution of correct and incorrect classifications in terms of the following:

- **True Positives** *(TP):* Correctly predicted positive cases;
- **True Negatives** *(TN):* Correctly predicted negative cases;
- **False Positives** *(FP):* Incorrectly predicted positive cases;
- **False Negatives** *(FN):* Incorrectly predicted negative cases.

The performance metrics were calculated using the following formulas:

$$\mathrm{Accuracy}\;=\;\frac{\left(TP+TN\right)}{\left(TP+\;FP+TN+FN\right)};$$

$$\mathrm{Precision}=\frac{TP}{(TP+FP)\;};$$

$$\mathrm{Recall}=\frac{TP}{(TP+FN)\;\;};$$

$$F1-\mathrm{Score}=\frac{\left(2\times Pression\times Recall\right)}{\left(Pression+Recall\right)}$$

Accuracy provides an overall correctness measure, while Precision and Recall are particularly important in imbalanced datasets, as they reflect the model’s performance in detecting minority class cases. The F1-score balances both precision and recall, offering a single metric that considers both false positives and false negatives, which is crucial in medical diagnostics where both types of errors have serious consequences. Additionally, receiver operating characteristic (ROC) curves evaluate classification thresholds.

## 4. Results

### 4.1. Baseline Model Performance on Raw Dataset

To evaluate the improvement achieved through our proposed preprocessing and feature selection pipeline, we first trained XGBoost and LightGBM classifiers on the raw dataset with all eight features (baseline). Next, structured preprocessing steps, including mean imputation, outlier removal, and random oversampling, were applied while still retaining all eight features. The performance summary before and after preprocessing is shown in Table 5. Both classifiers demonstrated substantial gains in accuracy, recall, and F1-score after preprocessing, confirming the effectiveness of the data cleaning and balancing strategy.

### 4.2. Feature Selection Strategy

#### 4.2.1. SHAP-Based Feature Importance Analysis

**Figure 7:**
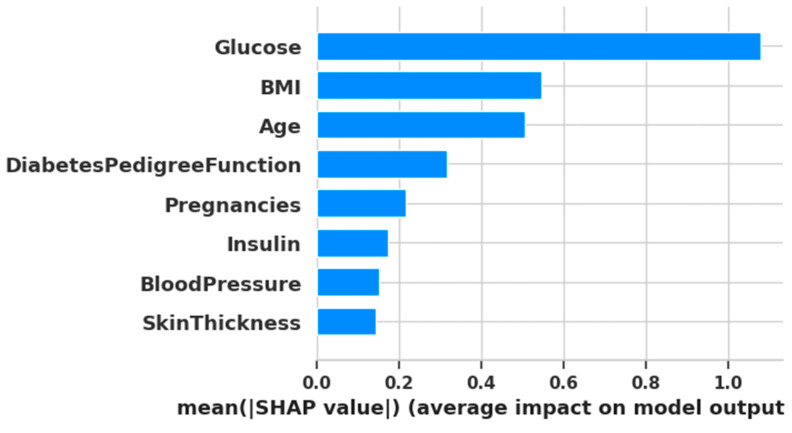
Plotting of SHAP analysis.

To gain an interpretable understanding of feature contributions, SHAP (Shapley Additive exPlanations) was used. A gradient boosting classifier was trained on the fully preprocessed and balanced dataset (via Random Oversampling). SHAP values were then calculated for all eight features. The resulting mean absolute SHAP values, representing feature importance, are visualized in Figure 7.

#### 4.2.2. Algorithm-Based Feature Selection

Initially, the parameters used for each algorithm are presented in Table 6.

Now, the summary Table 7, includes each algorithm’s average accuracy, F1, recall, number of features and the most frequent selected feature indices:

#### 4.2.3. Comparative Analysis of Feature Selection Approaches

To validate the reliability and consistency of the selected features, we compared the outputs from both SHAP-based feature importance analysis and algorithm-based feature selection methods.

From the SHAP-based ranking, the top six features based on mean SHAP values were: [1, 5, 7, 6, 0, 4] corresponding to Glucose, BMI, Age, Diabetes Pedigree Function, Pregnancies, and Insulin. On the other hand, algorithm-based selection methods most frequently selected the following features across five different algorithms:

- Feature 1 (Glucose)—selected by all algorithms;
- Feature 5 (BMI)—selected by four algorithms;
- Feature 6 (Diabetes Pedigree Function)—selected by four algorithms;
- Feature 0 (Pregnancies)—selected by three algorithms;
- Feature 2 (Blood Pressure)—selected by three algorithms;
- Feature 3 (Skin Thickness)—selected by three algorithms;
- Feature 7 (Age)—selected by three algorithms.

This comparison reveals a substantial overlap between the two approaches. Notably, features 1 (Glucose), 5 (BMI), and 6 (Diabetes Pedigree Function) appeared consistently across both SHAP and algorithm-based methods. This indicates their high predictive importance and suggests they should be retained in the final model.

### 4.3. Final Model Performance

In this part, the preprocessed dataset is divided using a 70–30 Split. Then, using the selected features from different selection methods, the two classification models are trained. Their performances are shown in Table 8.

**Figure 8:**
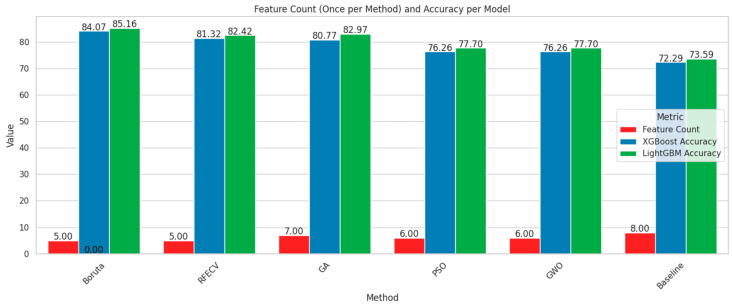
Comparison chart based on feature number and accuracy.

**Figure 9:**
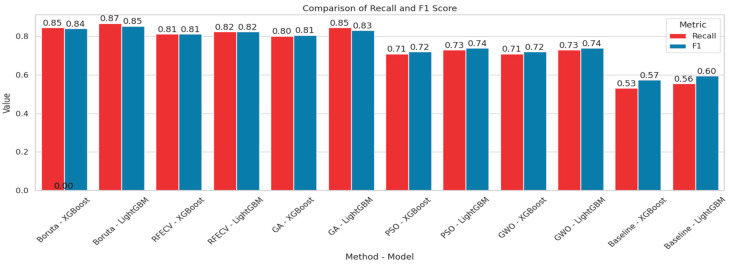
Comparison chart based on Recall and F1-score.

In order to visualize the comparison between the baseline models and the models after feature selection, bar plots have been generated (Figure 8 and Figure 9). These plots illustrate how different feature selection algorithms (Boruta, RFE, GA, PSO, GWO) affect model performance across key metrics such as the number of selected features, accuracy, recall and F1-score. This visual representation helps to identify which feature selection techniques clearly lead to improved or degraded performance.

**Figure 10:**
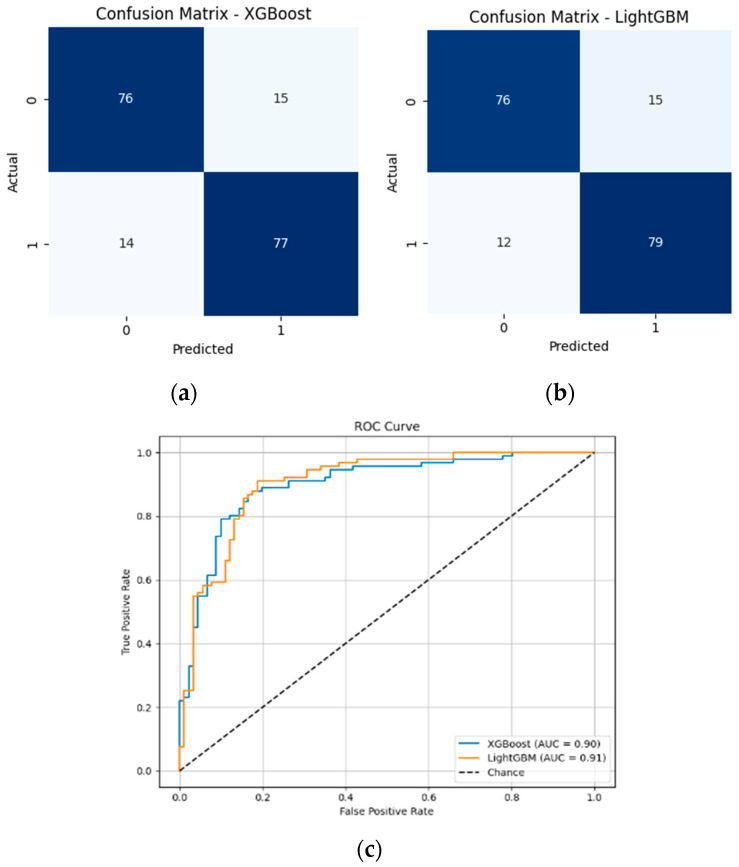
(**a**) Confusion matrix of XGBoost model with 5 features; (**b**) Confusion matrix of LGBM model with 5 features; (**c**) ROC curves of XGBoost and LGBM models with 5 features.

Among the feature selection methods evaluated, Boruta consistently achieved the best balance between reducing the number of features and maintaining or improving model performance. With only five selected features, Boruta-enabled models, especially LGBM, delivered the highest accuracy, recall, and F1-scores compared to other methods. For early diabetes diagnosis, features like Glucose, BMI, Age, Diabetes Pedigree Function, and Skin Thickness are particularly significant. To visualize the performances, corresponding confusion matrices and ROC curves are added in Figure 10.

Both XGBoost and LightGBM performed well after full preprocessing and Boruta-based feature selection. LightGBM achieved the highest accuracy (85.16%), F1-score (85.41%), and AUC (0.9052). Although XGBoost also performed competitively, LightGBM is preferred here due to its overall better balance of performance and efficiency.

To evaluate the impact of feature selection on model efficiency, we measured the training time for both the baseline model, which uses all 8 features without preprocessing, and our proposed model, which uses 5 features selected by Boruta. The summary is shown in Table 9.

The LightGBM model showed a significant gain, reducing training time from 0.0564 s to 0.0254 s while improving accuracy. Similarly, XGBoost benefited from feature reduction via Boruta, cutting the number of features from 8 to 5, which led to a 36.29% increase in F1-score (from 0.6174 to 0.8415) and an 81.94% reduction in training time (from 0.2246 s to 0.0406 s). This confirms that our reduced-feature pipeline not only enhances classification performance but also offers practical computational savings suitable for real-time or embedded health monitoring systems.

### 4.4. Validation on an Additional Dataset

To validate our proposed framework, we implemented the complete pipeline on the DiaHealth dataset. This involved several key steps:

- Numeric conversion with imputation for missing values;
- Outlier removal using the interquartile range (IQR) method;
- Class balancing via the Random Over-Sampling method;
- Feature selection using Boruta across 5-fold cross-validation;
- The final model performance was evaluated using LightGBM.

Now, Table 10 summarizes the performance of the baseline model and the final LightGBM model on the DiaHealth dataset, including the number of features used in each case, and also provides a comparison with the results reported in recent studies on this dataset.

Here, U.F.N. indicates the number of features used in the implementation.

From the table summary, it is evident that the TIPNet deep model [54] achieved moderate performance on the DiaHealth dataset using all 14 features. The baseline LightGBM classifier, trained on the raw dataset without preprocessing, achieved high accuracy but very low recall and F1-score, indicating poor detection of the minority class. After applying our complete pipeline, including preprocessing, class balancing, and Boruta-based feature selection, the final LightGBM model using only 10 selected features achieved outstanding performance, highlighting the effectiveness of our approach in both reducing dimensionality and improving minority class detection.

## 5. Discussion, Limitation, and Future Work

The present study showed that integrating robust preprocessing with Boruta-based feature selection significantly improves both predictive accuracy and computational efficiency for early diabetes detection. With only five features out of eight, the LightGBM classifier achieved an accuracy of 85.16% and an F1-score of 0.8541, while reducing training time by nearly 55% compared to the baseline. Similarly, XGBoost benefited from the reduced-feature set, with its training time decreasing by more than 80%, resulting in higher accuracy and a good F1-score. These results emphasize that the proposed framework not only enhances predictive performance but also offers substantial computational savings, making it particularly suitable for real-world healthcare applications where both accuracy and efficiency are important.

When compared to prior studies, our framework consistently outperforms traditional classifiers and ensemble methods. For example, Naïve Bayes with K-medoids undersampling on the CPCSSN dataset achieved 79% ROC [16], and Naïve Bayes on the PIDD reached 76.3% accuracy [17]. Random Forests also reported strong performance with an error rate of 0.21 [18], but all of these results fall short of our 85.16%. Similarly, an analysis using the UCI dataset reached 82.3% accuracy with Naïve Bayes and 11 out of 15 features [19], whereas our model achieves higher accuracy with fewer features (5/8), showing a more efficient approach. Feature selection methods like R-HEFS with Random Forests [20] yielded 73.04% accuracy with 3/8 features, demonstrating the importance of feature reduction but underperforming relative to Boruta. Other studies using boosting algorithms, such as improved XGBoost with feature engineering (80.2%) [21] and XGBoost with ADASYN augmentation (81%) [24], also report lower accuracy than our framework and do not emphasize training efficiency. Models based on kNN [22,27], logistic regression bagging [23], or Random Forests [26] reported accuracy levels in the 76–82% range, further underscoring the superiority of our proposed pipeline.

A key differentiating factor is that most prior works either used all available features [17,18,22,26,27] or relied heavily on data augmentation methods such as oversampling [16,24,28]. While these approaches improve class balance, they introduce risks of overfitting and increase computational burden. In contrast, our study shows that strategic feature reduction (from 8 to 5) combined with boosting classifiers not only enhances predictive power but also ensures interpretability and efficiency. The SHAP analysis confirmed that the selected features (Glucose, BMI, Age, Diabetes Pedigree Function, and Skin Thickness) are clinically relevant, aligning with known diabetes risk factors, thereby strengthening the medical interpretability of our results.

The validation of our proposed framework on the DiaHealth dataset [32] further demonstrates the robustness and generalizability of our approach. Despite being a recently released dataset with limited prior studies, our pipeline—including preprocessing, class balancing, and Boruta-based feature selection—enabled the LightGBM model to achieve outstanding performance, with 99.39% accuracy, 100% recall, and 99.39% F1-score. These results not only highlight the effectiveness of the framework in capturing relevant patterns and detecting the minority class but also show that it outperforms the recent TIPNet deep learning model [54], which achieved only 87% accuracy and 86% F1-score. The success on this secondary, real-world dataset underscores the potential of our methodology for early and reliable diabetes prediction, even in novel clinical datasets where existing studies are limited.

While the proposed framework demonstrates strong predictive performance on both the Pima Indian Diabetes Dataset (PIDD) and the recently released DiaHealth dataset, several limitations must be acknowledged. The PIDD is relatively small and demographically narrow, lacking longitudinal information such as time from initial diagnosis and the presence of comorbidities (e.g., vascular disease, hypertension), which are known to influence clinical parameters, including BMI and blood pressure. Consequently, the models may not fully capture the progressive nature of diabetes in real-world patients. Insulin levels, though clinically significant, were excluded from the final feature set due to a high proportion of missing values. Although mean imputation was applied for transparency and reproducibility, this approach may have reduced the predictive contribution of insulin. Moreover, the preprocessing pipeline—mean imputation and IQR-based outlier filtering—while reproducible and straightforward, removed a substantial number of outliers, raising concerns about external validity. Finally, the study relies on structured clinical features, without incorporating other complex data types such as imaging or genetic markers, which limits the scope of predictive insight. Despite the high performance achieved on the DiaHealth dataset, generalizability to broader populations and multi-center datasets remains to be fully evaluated.

Future efforts in this field can build upon our framework to further improve predictive performance, interpretability, and clinical utility. Sophisticated resampling techniques, including SMOTE, ADASYN, and GAN-based synthetic data augmentation, may mitigate class imbalance while reducing the risk of overfitting. Extending cross-validation to the entire model pipeline, rather than limiting it to feature selection, will ensure more robust and reliable performance evaluation. The inclusion of additional classifiers, such as Random Forest, Extra Trees, and other ensemble methods, can provide complementary perspectives and strengthen generalizability. Enhanced or adaptive versions of metaheuristic feature selection algorithms (PSO, GWO, GA) may optimize feature subsets more effectively. Furthermore, integrating advanced deep learning architectures (CNNs, transformers, autoencoders) and additional Explainable AI techniques could uncover complex feature relationships and improve interpretability. Finally, validating these approaches on longitudinal, multi-center, and demographically diverse datasets, combined with the development of real-time predictive systems, will be essential for translating machine learning models into practical clinical tools and for understanding diabetes progression over time.

## 6. Conclusions

In conclusion, this study successfully developed and evaluated an integrated machine learning framework for the early prediction of Type 2 Diabetes Mellitus using the Pima Indian Diabetes Dataset (PIDD). Addressing critical gaps in existing literature, our framework emphasized robust data preprocessing, advanced feature selection, and interpretable model evaluation. Key findings demonstrate that the proposed feature reduction framework, utilizing BorutaPy, effectively identified a highly informative subset of 5 features from the original 8, significantly improving accuracy from 73.59% to 85.16% using the LGBM algorithm. Along with enhancing performances, the training time also reduced 54.96% (from 0.0564 s to 0.0254 s) for the LGBM classifier. These findings highlight the potential of the proposed framework as a reliable diagnostic aid, especially in resource-constrained environments.

Furthermore, the framework was validated on the recently released DiaHealth dataset, which contains real-world clinical data and has limited prior studies. Our pipeline enabled the LGBM model to achieve outstanding performance with 99.39% accuracy, 100% recall, and 99.39% F1-score. These results underscore the robustness and generalizability of the proposed approach, demonstrating its effectiveness across multiple datasets and its superiority over recent models. Overall, this study contributes to the growing evidence that interpretable, feature-efficient machine learning models can offer clinically valuable insights for early diabetes detection.

## Figures and Tables

**Table 1:** Summary of literature review of various machine learning approaches for diabetes detection.

Ref. No	Dataset	Method	Key Findings	N.F.U	Year
[16]	Canadian Primary Care Sentinel Surveillance Network (CPCSSN)	DT, NB	Showed the supremacy of NB with the K-medoids undersampling technique as compared to random undersampling, oversampling, and no sampling, with an achievement of 79% receiver operating characteristic performance.	--	2018
[17]	PIDD	DT, SVM, and NB	Naïve Bayes outperforms with the highest accuracy of 76.30% compared to other algorithms.	8/8	2018
[18]	PIDD	RF	The random forest gave better performance with an error rate of 0.21 at 40 trees than any other method.	8/8	2019
[19]	UCI Diabetes Dataset	SVM, RF, NB, DT, and KNN	Naïve Bayesian outcome states the best accuracy of 82.30%.	11/15	2019
[20]	PIDD	Parameter-free greedy ensemble approach and RF	Uses Tenfold cross-validation and an accuracy of 73.04%.	3/8	2021
[21]	PIDD	Feature extraction with XGBoost	The improved XGBoost algorithm with feature combination is 80.2%	F. E.	2020
[22]	PIDD	Ensemble of multiple classifiers, including DT, NB, KNN, LR, etc.	With the proposed algorithm, accuracy has risen from 70.1% to 78.58%, which is an increase of 8.48%	8/8	2021
[23]	PIDD	K-means feature selection and Bagging (LR)	Tenfold cross-validation and accuracy of 82.00%.	8/8	2022
[24]	PIDD and RTML	XGBoost, RF, SVM, KNN, LR, DT, AdaBoost, Bagging, Voting	XGBoost with ADASYN achieved the highest accuracy of 81% (AUC 0.84) on the merged dataset; 96% accuracy on the private RTML dataset with domain adaptation. Mutual information identified Glucose, BMI, Age, and Insulin as key features.	8/8	2022
[25]	PIDD	KNN, BNB	The KNN model achieved the highest accuracy in detecting diabetes, with 79.6%, surpassing the BNB model’s 77.2%.	8/8	2023
[26]	PIDD	RF, DT, NB, and LR.	Results of the study showed that RF performs better with an accuracy of 80%, precision of 82%, error rate of 20%, and sensitivity of 88% in comparison to other developed models, DT, NB, and LR	8/8	2024
[27]	PIDD	KNN	The KNN model achieved an accuracy of 76%, with a precision of 0.80, a recall of 0.85, an F1-score of 0.83, and support of 167 for the test set.	8/8	2024
[28]	PIDD	SMOTE with C5.0, RF, SVM	It can be inferred that there is minimal impact post-SMOTE across the three classification models due to potential overfitting on the dataset.	8/8	2024

N.F.U. indicates the number of features used in that work, and F. E. refers to feature engineering, -- indicates unspecified feature number.

**Table 2:** Attribute-wise Count, Minimum, Maximum, Mean, and Standard Deviation of PIDD Features.

Feature No.	Feature Name	Count	Min	Max	Mean	Std Dev
0	Pregnancies	768	0.0	17.0	3.84	3.36
1	Glucose		0.0	199.0	120.89	31.97
2	BloodPressure		0.0	122.0	69.10	19.35
3	SkinThickness		0.0	99.0	20.53	15.95
4	Insulin		0.0	846.0	79.79	115.24
5	BMI		0.078	67.1	31.99	7.88
6	DiabetesPedigreeFunction		21.0	2.42	0.47	0.33
7	Age		0.0	81.0	33.24	11.76

**Table 3:** Features of the DiaHealth dataset, including attribute names, data types, and brief descriptions of their clinical or demographic relevance to Type 2 Diabetes prediction.

Feature No.	Feature Name	Description	Data Type
0	age	Patient’s age in years	int64
1	gender	Patient’s biological indicator	object
2	pulse_rate	Heart rate	int64
3	systolic_bp	Systolic blood pressure	int64
4	diastolic_bp	Diastolic blood pressure	int64
5	glucose	Glucose concentratyion of blood	float64
6	height	Patient’s height	float64
7	weight	Patient’s weight	float64
8	bmi	Patient’s body mass index	float64
9	family_diabetes	Family history about diabetes	int64
10	hypertensive	Presence of hypertension	int64
11	family_hypertension	Family history of hypertension	int64
12	cardiovascular_disease	Presence of cardiovascular diseases	int64
13	stroke	History of stroke	int64
14	diabetic	Target variable	object

**Table 4:** Number of missing values per feature.

Feature Name	Missing Values per Column
Pregnancies	0
Glucose	5
BloodPressure	35
SkinThickness	227
Insulin	374
BMI	11
DiabetesPedigreeFunction	0
Age	0

**Table 5:** Performance of XGBoost and LightGBM before and after preprocessing.

Model	Accuracy (Baseline)	Accuracy (After Preprocessing)	Recall (Baseline)	Recall (After Preprocessing)	F1-Score (Baseline)	F1-Score (After Preprocessing)
XGBoost	0.7532	0.8242	0.5679	0.8242	0.6174	0.8242
LightGBM	0.7359	0.8242	0.5556	0.8352	0.5960	0.8253

**Table 6:** Parameters used for feature selection algorithms.

Algorithm	Hyperparameters
Boruta	n_estimators = auto, random_state = 42, cross-validation = 5-fold StratifiedKFold
RFE	step = 1, cv = 5-fold, min_features_to_select = 1
GA	population_size = 20, generations = 15, mutation_rate = 0.01, selection = tournament (size = 3), crossover = single-point, cv = 5-fold
PSO	population = 10, max_iter = 15, w = 0.72, c1 = 1.5, c2 = 1.5, encoding = binary (0/1), cv = 5-fold
GWO	population = 10, max_iter = 15, selection threshold > 0.5, fitness = accuracy, cv = 5-fold

**Table 7:** Feature selection algorithm’s average accuracy, F1-score, recall, number of features, and the most frequent selected feature indices summary.

Algorithm	Avg Accuracy	Avg F1-Score	Avg Recall	Number of Features (Most Frequent)	Most Frequent Feature Indices
BORUTA	0.7964	0.8058	0.8478	5	[1, 3, 5, 6, 7]
RFE	0.7169	0.7079	0.6952	5	[0, 1, 2, 5, 6]
GA	0.8179	0.8235	0.8546	7	[0, 1, 2, 3, 4, 6, 7]
PSO	0.7981	0.8037	0.8313	6	[0, 1, 2, 3, 5, 7]
GWO	0.7948	0.8032	0.8445	6	[1, 2, 3, 5, 6, 7]

**Table 8:** Final Classification results using LGBM and XGBoost methods using selected features.

Feature Selection Method	No. of Features	Model	Accuracy (%)	Precision (Macro Avg)	Recall (Macro Avg)	F1-Score (Macro Avg)
Boruta	5	XGBoost	84.07	0.8370	0.8462	0.8415
		LightGBM	85.16	0.8404	0.8681	0.8541
RFECV	5	XGBoost	81.32	0.8138	0.8132	0.8131
		LightGBM	82.42	0.8244	0.8242	0.8242
GA	7	XGBoost	80.77	0.8111	0.8022	0.8066
		LightGBM	82.97	0.8191	0.8462	0.8324
PSO	6	XGBoost	76.26	0.7400	0.7100	0.7200
		LightGBM	77.70	0.7600	0.7300	0.7400
GWO	6	XGBoost	76.26	0.7400	0.7100	0.7200
		LightGBM	77.70	0.7600	0.7300	0.7400

**Table 9:** Summary of performance with training time for baseline performance vs. performance with the proposed framework.

Model	Features Used	Preprocessing	Accuracy (%)	F1-Score	Training Time (s)
XGBoost	8 (all)	NO	75.32	0.6174	0.2246
LightGBM	8 (all)	NO	73.59	0.5960	0.0564
XGBoost	5 (Boruta)	YES	84.07	0.8415	0.0406
LightGBM	5 (Boruta)	YES	85.16	0.8541	0.0254

**Table 10:** Performance comparison of baseline and final LightGBM models on the DiaHealth dataset, including the number of features used (U.F.N.), and comparison with the recent TIPNet deep learning model [54].

Model	Used Framework	U.F.N.	Accuracy	Recall	F1-Score
[54]	TIPNet Deep Model	14	87%	88%	86%
Baseline	LGBM classifier on raw (no preprocessing) dataset	14	94.49%	29.13%	40.00%
Final	UsedLGBM classifier on Boruta-selected features from preprocessed dataset	10	99.39%	100.00%	99.39%

## Data Availability

The dataset is available in the Mendeley Data Repository (dataset name: Pima Indians Diabetes Dataset (PIDD), DOI:10.17632/7zcc8v6hvp.1, https://data.mendeley.com/datasets/7zcc8v6hvp/1, accessed on 7 September 2025.

## Abbreviations

The following abbreviations are used in this manuscript:

RFE	Recursive Feature Elimination
GWO	Grey Wolf Optimizer
PSO	Particle Swarm Optimizer
GA	Genetic Algorithm
CV	Cross-Validation
LGBM	Light Gradient Boosting Machine Algorithm
XGBoost	Extreme Gradient Boosting Algorithm
